# Characteristics associated with decrements in objective measures of physical function in older patients with cancer during chemotherapy

**DOI:** 10.1007/s00520-022-07416-5

**Published:** 2022-11-03

**Authors:** Ann Helen Torstveit, Christine Miaskowski, Borghild Løyland, Ellen Karine Grov, Christine Seel Ritchie, Steven M. Paul, Anna Marie Ellström Engh, Inger Utne

**Affiliations:** 1 Department of Nursing and Health Promotion, Faculty of Health Sciences, OsloMet-Oslo Metropolitan University, Pilestredet 32, Oslo, Norway; 2 School of Nursing, University of California, San Francisco, CA USA; 3 Division of Palliative Care and Geriatric Medicine, Massachusetts General Hospital, Mongan Institute Center for Aging and Serious Illness, Boston, MA USA; 4 Department of Obstetrics and Gynecology, Akershus University Hospital, Lørenskog, Norway; 5 Faculty of Medicine, University of Oslo, Oslo, Norway

**Keywords:** Balance, Cancer, Chemotherapy, Gait speed, Older patients, Physical function

## Abstract

**Purpose:**

Study purposes were to evaluate for inter-individual variability in the trajectories of three objective measures of physical function (PF) in older patients receiving chemotherapy (*n* = 112) and determine which characteristics were associated with worse PF.

**Methods:**

Balance, gait speed, and chair-stand test were evaluated at initiation and 1, 3, 6, 9, and 12 months following chemotherapy. Hierarchical linear modeling was used to assess inter-individual variability in the trajectories of the three tests. Demographic, clinical, and symptom characteristics, and levels of cognitive function associated with initial levels and changes over time in each of the tests were determined.

**Results:**

Gait speed and chair-stand tests improved over time. Balance declined until month 6, then increased. Characteristics associated with decreases in balance scores at initiation of chemotherapy were lower level of education and lower Karnofsky Performance Status (KPS) score. For initial levels of poorer gait speed, older age, poorer Trail Making Test B (TMTB), and worse Attentional Function Index scores were the associated characteristics. Lower KPS scores, higher body mass index, and poorer TMTB scores were associated with poorer chair-stand times at initiation of chemotherapy. Worse trajectories of chair-stand times were associated with poorer chair-stand time at enrollment. Characteristic associated with lower initial levels and improved trajectories of balance was older age at enrollment.

**Conclusions:**

Determination of characteristics associated with decrements in balance, gait speed, and chair-stand can assist clinicians to identify older oncology patients at risk for decrements in PF. Interventions to maintain and improve PF need to be implemented with higher risk patients.

**Supplementary Information:**

The online version contains supplementary material available at 10.1007/s00520-022-07416-5.

## Introduction

Decrements in physical function (PF) in older oncology patients are associated with increased health care utilization and shorter survival [1]. While chemotherapy is a common treatment, limited information is available on its impact on PF in older adults during and following its completion. Most of the studies of PF in older oncology patients used self-report measures. However, recent findings suggest that subjective and objective measures of PF assess different dimensions of functional status [2]. Of ten longitudinal studies of PF in older patients undergoing chemotherapy [3–12], only five used objective measures and findings were inconsistent [8–12]. In one that evaluated for changes in grip strength, chair-stand, and 2-min walk tests, over 1 year, in 97 older patients with acute myeloid leukemia [10], grip strength showed an initial decline with subsequent recovery, the other two tests improved over time. In another study of 38 older patients [9], grip strength declined, chair-stand scores remained stable, and walking distance improved from initiation to third cycle of chemotherapy.

In the third study of 30 older patients with lung cancer [8], walk speed decreased from prior to through weeks 6 and 12 after chemotherapy administration. In the fourth study that evaluated for changes in PF in 32 patients with multiple myeloma at diagnosis and 6 months later [12], no differences in Timed-Up-and-Go test scores were found. In the fifth study of 49 patients who were recruited before or within 3 days of starting chemotherapy treatment for leukemia [11], compared to pretreatment scores, independent activities of daily living (ADL), Short Physical Performance Battery (SPPB) test scores, and grip strength declined at 8 weeks after hospital discharge. While these studies provide objective data on changes in PF, none evaluated whether selected demographic, clinical, and symptom characteristics were associated with decrements in PF. Further, four studies had relatively small sample sizes and short-term follow-up [8, 9, 11, 12]. Equally important, while these longitudinal studies used objective tests from the SPPB [8–12], only one of them used all three SPPB tests (i.e., balance, gait speed (GS), and chair-stand) [11]. As noted previously [13], each of the SPPB tests provides unique information about PF.

While none of these longitudinal studies determined risk factors associated with decreases in PF [8–12], findings from cross-sectional studies of older adults suggest that different factors are associated with objective measures of PF. For example, poorer balance was associated with older age [14], more pain and depression [15, 16], and lower cognitive function (CF) [17]. Reviews suggest that declines in PF and CF are connected and frequently co-occur in older adults [18, 19]. In contrast, decrements in GS were related to older age [14], lower levels of education [20], higher comorbidity burden [21], more pain and depression [15, 22], and decrements in CF [23]. Prolonged chair-stand times were associated with older age [14]. In our previous study [2], balance, GS, and chair-stand test scores exhibited small to moderate correlations (i.e., correlations between GS and balance *r* = 0.36, *p* < 0.001; GS and chair-stand *r* = 0.23, *p* = 0.009; chair-stand and balance *r* = 0.10, *p* = 0.275). These findings suggest that the three tests measure distinct, but related, dimensions of PF.

Given the paucity of research on changes in and risk factors for decrements in PF in older oncology patients, the purposes of this study were to evaluate for inter-individual variability in the trajectories of three objective measures of PF (i.e., balance, GS, and chair-stand) and determine which demographic, clinical, and symptom characteristics, as well as measures of PF and CF were associated with decrements in each test. Based on our previous findings [2] and those of others [14–17, 20–23], we hypothesized that different demographic, clinical, and symptom characteristics as well as PF and CF scores would be associated with changes in balance, GS, and chair-stand tests.

## Materials and methods

A detailed description of the methods is presented in Appendix A and in previous publications [24, 25].

### Sample and settings

In brief, eligible patients were ≥ 60 years of age; had a diagnosis of gynecological or colorectal cancer; were scheduled to receive chemotherapy; had a Montreal Cognitive Assessment (MoCA) score of ≥ 23 [26]; and had a Karnofsky Performance Status (KPS) score of ≥ 60 [27]. A total of 208 patients were approached and 149 consented to participate. Of these, one withdrew and nine were excluded because of low MoCA score. Complete data from 112 patients were available.

### Instruments

Patients completed a demographic questionnaire, the KPS scale that ranged from 40 to 100 [27], and the Self-Administered Comorbidity Questionnaire (SCQ-16) that ranged from 0 to 48 [28]. Balance score (from 0 to 4 points), timed GS (m/s), and the timed 5-times chair-stand test (s) from the SPPB were used to assess PF [13]. For balance, a total score of ≥ 3.71 (± 0.65) is considered normal [14]. For GS and chair-stand tests, < 1.2 m/s (i.e., MCID for gait speed = 0.05 m/s) and ≤ 11.19 s, respectively, are considered normal.

Center for Epidemiological Studies-Depression (CES-D) scale was used to evaluate depressive symptoms, with scores ≥ 16 indicating need for clinical evaluation. Additional symptoms were assessed using European Organization for the Research and Treatment of Cancer Quality of Life Questionnaire Core-30 (i.e., QLQ-C30). CF was assessed using the self-reported Attentional Function Index (AFI) [29], which is grouped into three categories (< 5.0 low function, 5.0 to 7.5 moderate function, > 7.5 high function), and the MoCA test [26], with scores ≤ 25 indicating cognitive impairments, and the timed Trail Making Test B (TMTB test) (seconds) [30].

### Study procedures

Regional Committee for Medical and Research Ethics, Norway and the Institutional Review Board at each of the study sites approved the study (reference No. 2015/1277/REC South-East). Clinicians approached patients prior to the initiation of chemotherapy to assess their interest in study participation. Written informed consent was obtained from all patients. Patients completed study questionnaires and PF tests in their homes or in the clinic, prior to and 1, 3, 6, 9, and 12 months after the initiation of chemotherapy. Reliability testing was done on an annual basis. An inter-rater reliability of > 0.90 was achieved for the study measures.

### Statistical analysis

Descriptive statistics were generated for demographic and clinical characteristics, symptom severity scores, and measures of PF and CF using SPSS version 26 (IBM Corporation, Armonk, NY). Demographic, clinical, and symptom characteristics, as well as the CF measures that were evaluated as predictors in the hierarchical linear modeling (HLM) analysis were assessed at the initiation of chemotherapy.

HLM based on full maximum likelihood estimation was done using software developed by Raudenbush and Bryk [31]. Separate HLM analyses were done for balance, GS, and chair-stand tests. In brief, during stage 1, intra-individual variability in the scores for each test over time was examined. At this point, the model was constrained to be unconditional and likelihood ratio tests were used to determine the best fitting model.

Second stage of HLM analysis examined inter-individual differences in the trajectories of each test’s scores by modeling the individual change parameters as a function of proposed predictors at level 2. Supplemental Tables 1, 2, and 3 present the list of proposed predictors for each test. To construct a parsimonious model, exploratory level 2 analysis was completed and predictors with a *t* value of < 2.0 were dropped from subsequent model testing. Significant predictors from the exploratory analyses were entered into the model to predict each individual change parameter. Predictors that maintained a statistically significant contribution in conjunction with other variables were retained in the final model. A *p* value of < 0.05 indicated statistical significance.

## Results

**Fig. 1:**
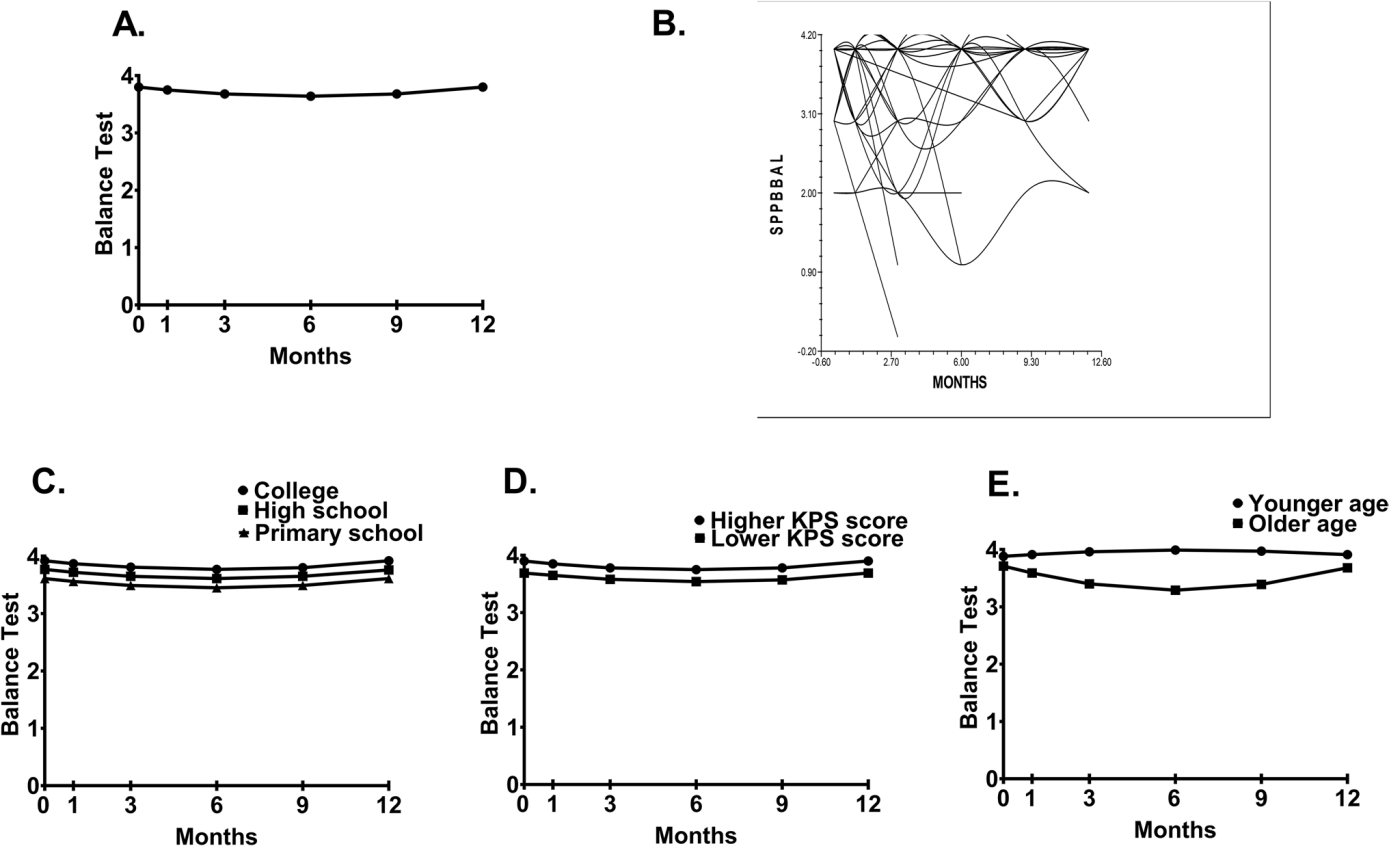
**A** Unconditional model of mean balance test scores at the initiation of chemotherapy and at 1, 3, 6, 9, and 12 months after its initiation. **B** Spaghetti plot of a random sample of 50% of the patients’ balance score trajectories over 12 months. Influence of enrollment scores for **C** education (i.e., primary school vs high school vs college), as well as **D** KPS score (lower/higher calculated as one SD above and below the mean KPS score), on inter-individual differences in the intercept for the balance test. Influence of the enrollment score for **E** age (i.e., younger/older calculated as one SD above and below the mean age) on inter-individual differences in the intercept and slope parameter for the balance test

**Fig. 2:**
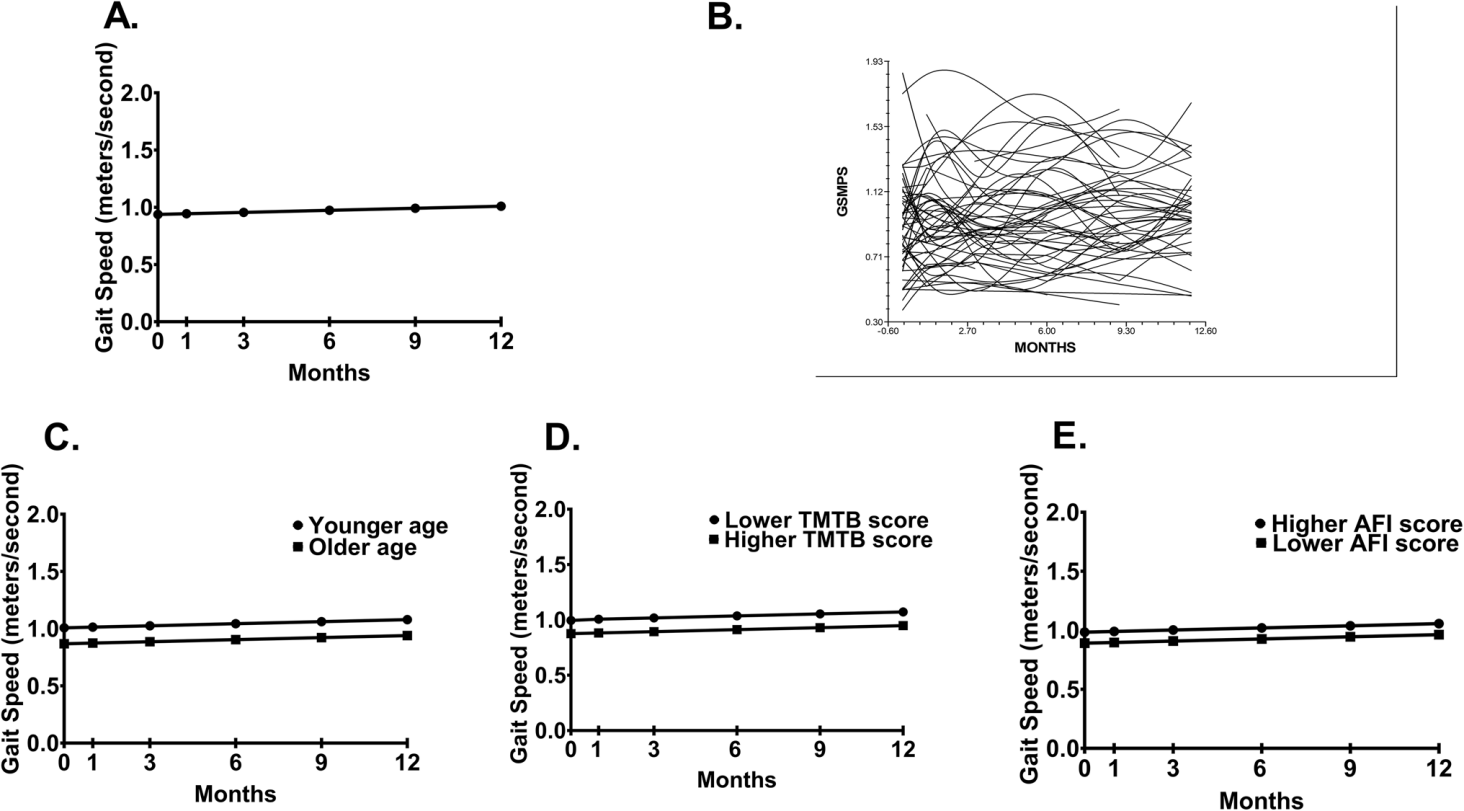
**A** Unconditional model of mean gait speed time at the initiation of chemotherapy and at 1, 3, 6, 9, and 12 months after its initiation. **B** Spaghetti plot of a random sample of 50% of the patients’ gait speed time trajectories over 12 months. Influence of enrollment scores for **C** age, **D** TMTB score, and **E** AFI score (i.e., younger/older and lower/higher calculated as one SD above and below the mean age, TMTB score and AFI score, respectively), on inter-individual differences in the intercept for gait speed time

**Fig. 3:**
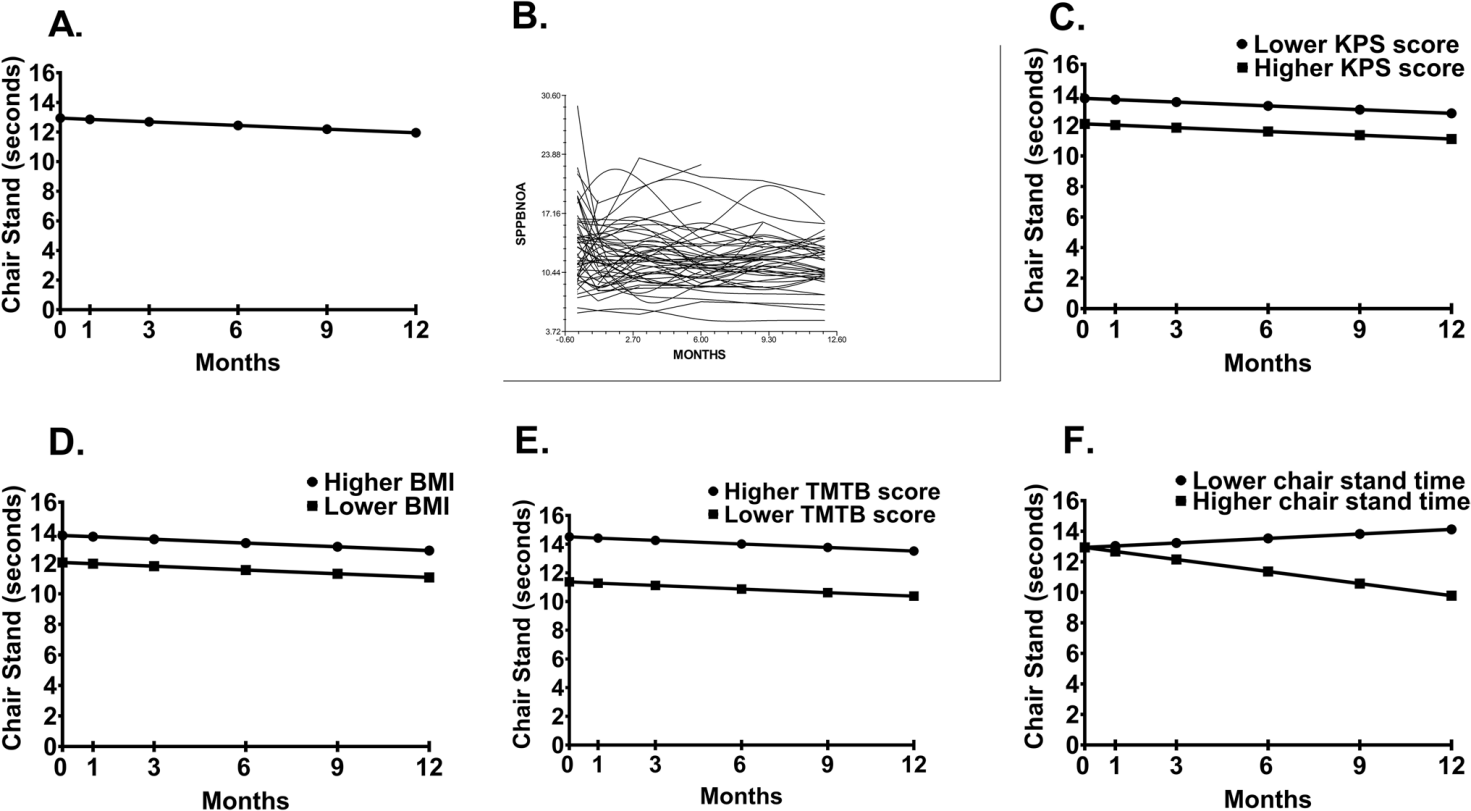
**A** Unconditional model of mean chair-stand time at the initiation of chemotherapy and at 1, 3, 6, 9, and 12 months after its initiation. **B** Spaghetti plot of a random sample of 50% of the patients’ chair-stand time trajectories over 12 months. Influence of enrollment scores for **C** KPS score, **D** BMI, and **E** TMTB score (i.e., lower/higher calculated as one SD above and below the mean KPS, BMI and TMTB scores), on the inter-individual differences in the intercept for chair-stand time. Influence on the score for **F** chair-stand time (i.e., lower/higher calculated as one SD above and below the mean chair-stand time) at enrollment on inter-individual differences in the slope parameter for chair-stand time

Figures 1D–E, 2C–E, and 3C–F display the adjusted change curves for the balance test, gait speed test, and chair-stand test, respectively, that were estimated based on one standard deviation (SD) above and below the mean score of the predictor variables.

### Patient characteristics

As reported [25], patients (*n* = 112) were 70.4 (± 6.5) years of age, well-educated, and diagnosed with gynecological (90.2%) or colorectal (9.8%) cancer. Most patients were female (93.8%), married (64.3%), and not employed (83.0%). Mean number of comorbidities was 2.0 (± 1.7) and mean SCQ score was 3.8 (± 3.8). The patients were 1.3 (± 3.9) years from their cancer diagnosis, had metastatic disease (78.6%), and had surgery prior to chemotherapy (54.5%; Table 1).

**Table 1:** Demographic, clinical, and symptom characteristics of the sample at enrollment (*n* = 112)

Demographic and clinical characteristic	Mean (SD^a^)
Age (years)	70.4 (6.5)
Time since cancer diagnosis (years)	1.3 (3.9)
Karnofsky Performance Status score	87.1 (10.1)
Body mass index	26.2 (6.3)
Hemoglobin (grams/deciliter)	12.6 (1.7)
Number of comorbid conditions out of 16	2.0 (1.7)
Self-Administered Comorbidity Questionnaire score	3.8 (3.8)
	*n* (%)
Female gender (% yes)	105 (93.8)
Education	
Primary school	18 (16.1)
High school	54 (48.2)
College	40 (35.7)
Cancer diagnosis	
Gynecological	101 (90.2)
Colorectal	11 (9.8)
Married or partnered (% yes)	72 (64.3)
Lives alone (% yes)	37 (33.0)
Currently employed (% yes)	19 (17.0)
Presence of metastatic disease (% yes)	88 (78.6)
Surgery prior to chemotherapy (% yes)	61 (54.5)
Symptom characteristics	Mean (SD)
Center for Epidemiologic Studies Depression Scale (CES-D) score	11.6 (8.2)
EORTC QLQ-C30^b^ fatigue score	43.9 (26.3)
EORTC QLQ-C30 nausea and vomiting score	8.0 (15.2)
EORTC QLQ-C30 dyspnea score	20.5 (28.7)
EORTC QLQ-C30 insomnia score	24.7 (29.3)
EORTC QLQ-C30 appetite loss score	26.8 (34.0)
EORTC QLQ-C30 constipation score	28.9 (33.6)
EORTC QLQ-C30 diarrhea score	11.3 (23.5)
EORTC QLQ-C30 pain score	33.3 (29.8)
Physical and cognitive function measures	Mean (SD)
Balance score	3.8 (0.5)
Gait speed (meters per second)	0.9 (0.3)
Chair-stand (seconds)	13.4 (5.5)
Attentional Function Index score	7.4 (1.5)
Montreal Cognitive Assessment score	26.3 (2.1)
Trail Making Test B (seconds)	112.5 (50.2)

^a^Standard deviation

^b^European Organization for the Research and Treatment of Cancer Quality of Life Questionnaire

At enrollment, the mean score on the CES-D (11.6 ± 8.2) was below the clinically meaningful cutoff score of ≥ 16. Mean scores on the QLQ-C30 symptom scales ranged from 8.0 (± 15.2) for nausea/vomiting to 43.9 (± 26.3) for fatigue. While enrollment score for chair-stand test of 13.4 (± 5.5) suggests lower level of PF, the scores for balance (3.8 ± 0.5), GS (0.9 ± 0.3), MoCA (26.3 ± 2.1), TMTB (112.5 ± 50.2), and AFI (7.4 ± 1.5) suggest normal levels of PF and CF (Table 1).

### Balance test

First stage of HLM analysis examined how balance scores changed from initiation of chemotherapy through 12 months. In the unconditional, quadratic model, the intercept represents the estimated balance score (i.e., 3.794 on a 0 to 4 scale) at enrollment. Estimated linear rate of change, for each additional assessment, was − 0.045 and the estimated quadratic change was 0.004 (*p* < 0.05; Table 2).

**Table 2:** Hierarchical linear model for the balance test, gait speed test, and chair-stand test

	Coefficient (SEe)	
	Unconditional model	Final model
**Balance test**		
Fixed effects		
Intercept	3.794 (0.041)^c^	3.797 (0.038)^c^
Time (months) (linear rate of change)	− 0.045 (0.019)^a^	− 0.052 (0.018)^b^
Time2 (months) (quadratic rate of change)	0.004 (0.002)^a^	0.004 (0.001)^b^
Time invariant covariates		
Intercept		
Age		− 0.013 (0.006)^a^
Karnofsky Performance Status score		0.010 (0.003)^b^
Education		0.157 (0.050)^b^
Linear		
Age		− 0.013 (0.003)^c^
Quadratic		
Age		0.001 (0.0003)^c^
Variance components		
In intercept	0.072^b^	0.045^d^
In linear slope	0.011^d^	0.005^d^
In quadratic slope	0.0001^d^	0.00002^d^
Goodness-of-fit deviance (parameters estimated)	826.184 (10)	777.829 (15)
Model comparison (*X*^2^)		48.355 (5)^c^
**Gait speed (meters per second)**		
Fixed effects		
Intercept	0.937 (0.023)^c^	0.937 (0.021)^c^
Time (months) (linear rate of change)	0.007 (0.002)^b^	0.006 (0.002)^b^
Time invariant covariates		
Intercept		
Age		− 0.011 (0.003)^b^
Trail Making Test B score		− 0.001 (0.0004)^b^
Attentional Function Index score		0.032 (0.014)^a^
Variance components		
In intercept	0.048^c^	0.039^c^
In slope	0.0002^c^	0.0002^c^
Goodness-of-fit deviance (parameters estimated)	− 203.942 (6)	− 238.434 (9)
Model comparison (*X*^2^)		34.492 (3)^c^
**Chair-stand (seconds)**		
Fixed effects		
Intercept	12.914 (0.389)^c^	12.936 (0.340)^c^
Time (months) (linear rate of change)	− 0.085 (0.037)	− 0.082 (0.030)^b^
Time invariant covariates		
Intercept		
Karnofsky Performance Status score		− 0.083 (0.033)^a^
Body mass index		0.141 (0.055)^a^
Trail Making Test B score		0.031 (0.007)^c^
Linear		
Chair-stand test score at enrollment		− 0.033 (0.055)^c^
Variance components		
In intercept	13.905^c^	9.891^c^
In slope	0.061^c^	0.012^d^
Goodness-of-fit deviance (parameters estimated)	2785.301 (6)	2737.808 (10)
Model comparison (*X*^2^)		47.493 (4)^c^

^a^*p* < 0.05

^b^*p* < 0.01

^c^ < 0.001

^d^Not significant

^e^Standard error

Balance scores decreased slightly until month 6 and then increased to month 12 (Fig. 1A). As noted by the variance components (Table 2) and seen in Fig. 1B, a considerable inter-individual variability existed in the balance score trajectories. These results supported analyses of predictors of inter-individual variability in initial levels, and the trajectories of these scores. Mean scores for all PF tests for the various groups depicted in the figures are estimated or predicted means based on the HLM analyses.

Second stage of the HLM analysis evaluated how balance scores at the initiation of chemotherapy and its change over time were associated with demographic, clinical, and symptom characteristics, as well as CF. Characteristics associated with inter-individual variability in balance scores at initiation of chemotherapy were education and KPS score (Table 2). As shown in Fig. 1C and D, patients with a lower level of education and a lower performance status were more likely to have poorer balance at enrollment. Age was the only characteristic that was associated with inter-individual variability in both initial levels and the trajectories of the balance scores (Fig. 1E). Patients who were older at enrollment had decreases in balance scores at the first three assessments and then increase in balance scores at the fifth and sixth assessments. In contrast, younger patients had higher and stable balance scores over time.

### Gait speed test

In the unconditional, linear model, the intercept represents the estimated GS (i.e., 0.937 m/s) at the initiation of chemotherapy (Table 2). The estimated linear rate of change in GS, for each additional assessment, was 0.007 (*p* < 0.01). As shown in Fig. 2A, GS increased from enrollment to month 12, but exhibited a large amount of inter-individual variability (Fig. 2B).

Characteristics associated with inter-individual variability in GS at initiation of chemotherapy were age, TMTB score, and AFI score. No characteristics were associated with inter-individual variability in its trajectory (Table 2). As shown in Fig. 2C to E, older age, higher TMTB scores, and lower AFI scores were associated with slower GS at the initiation of chemotherapy.

### Chair-stand test

In the unconditional, linear model, the intercept represents the estimated chair-stand time (i.e., 12.914 s) at enrollment (Table 2). The estimated rate of change in the chair-stand time for each additional assessment was 0.085 s (*p* < 0.05). As shown in Fig. 3A, while chair-stand time decreased from enrollment to month 12, it exhibited a significant amount of inter-individual variability (Fig. 3B).

Characteristics associated with inter-individual variability in chair-stand times at enrollment were KPS score, body mass index (BMI), and TMTB score (Table 2). As shown in Fig. 3C to E, patients with a lower performance status, as well as a higher BMI and higher TMTB scores were more likely to have worse chair-stand times. As illustrated in Fig. 3F, chair-stand time at enrollment was associated with inter-individual differences in its linear slope. Patients who had a slower chair-stand time at enrollment were more likely to have a faster chair-stand time over time. Patients who had faster chair-stand time at enrollment had a slower chair-stand time over time.

## Discussion

This study is the first to assess for inter-individual differences in balance, GS, and chair-stand times, as well as for characteristics associated with these differences in oncology patients with median age of 70.1 from initiation of chemotherapy through 12 months. Our a priori hypothesis that common and distinct characteristics would be associated with decrements in each of the tests was partially supported. While several different demographic and clinical characteristics, and CF measures, were associated with the various PF tests, no associations were found with any of the symptom severity scores. The remainder of the discussion focuses on each of the PF measures.

Our sample’s balance score at enrollment (3.8) was comparable to an age matched sample from the general Norwegian population (3.7) [14] and slightly higher (3.1) than those of older patients with leukemia prior to chemotherapy [11]. In terms of its trajectory, while our initial findings are consistent with a study of older patients with leukemia [11], our patients’ decrements in balance were relatively small (i.e., a change of 0.2 at 6 months) and not clinically meaningful.

In terms of risk factors, consistent with findings in the geriatric literature [14], older age was associated with worse balance scores at the initiation of chemotherapy, as well as a subsequent decline in balance over 6 months followed by a return to pretreatment levels at 12 months. Normal age-related decreases in postural control occur due to changes in muscle morphology, neuromuscular transmission [32], and reduced visuospatial and cognitive processing abilities [33]. These changes contribute to balance problems and increased risk for falls [34]. While no studies identified an association between lower levels of education and poorer balance in older patients with cancer, in previous research with older adults [35, 36], lower socioeconomic status was associated with decrements in PF. Given that, along with age, education and income are proxies for social determinants of health [37], future studies need to explore these relationships in more depth.

In our study, lower KPS score at the initiation of chemotherapy was associated with poorer balance. Our patients’ KPS scores (87.1) were consistent with previous studies of older patients with leukemia (83.4) [9], and a variety of solid tumors (82.6) [3]. While a KPS score of 80 to 90 suggests that patients have some symptoms of disease but can, with some effort, carry out normal activities, patients with a KPS score of 77.0 (i.e., 1 SD below the mean) would have problems carrying out normal work and activities [27]. While no studies evaluated the direct relationship between KPS scores and balance measures, our findings are congruent with another study that found that patients with chemotherapy-induced neuropathy who did not exercise had lower KPS and worse balance scores [38]. While age and education are non-modifiable risk factors, given the positive association between exercise and improvements in functional status [38] and balance [39], older patients with low KPS scores warrant referral to physical and occupational therapy.

Our patients’ GS time at enrollment (0.94 m/s) is significantly slower than age-matched normative data for Norwegian females (1.08, *t* = 25.1, *p* < 0.001) [14]. While our sample’s GS increased over time, it remained slower than the general population 12 months after enrollment (1.01). Findings regarding changes in GS in older patients with cancer are inconsistent, with some studies reporting increases [9, 10, 12] and others declines [8, 11]. This may be related to differences in mean age, cancer diagnoses, types of treatment, and stages of disease.

Older age, higher TMTB scores, and lower AFI scores were associated with poorer GS. GS is a complex task dependent on body composition and strength, energetics, and homeostatic regulation, as well as CF [40]. While the exact relationships between various brain functions and gait are not well understood, research suggests that higher levels of CF and memory are needed to organize mobility [41]. In addition, a prior study of older adults found associations between CF and PF [42]. As noted in one review [41], compared to other age-related changes, a decrease in gray matter had the strongest association with poorer gait. In addition, white matter atrophy, decline in hippocampal volume, ventricular enlargement, and amyloid and tau aggregation were associated with poorer GS. These associations may explain some of our findings.

While older age was associated with decrements in balance at enrollment and over time, its association with GS was found only at enrollment. Given that older age is associated with decrements in both balance and GS, two important measures of PF, and that decreases in GS are associated with impairments in ADL, reduced quality of life, and increased mortality [40], targeted interventions are warranted throughout chemotherapy treatment.

It is interesting to note that worse scores for both the objective and subjective measures of CF, namely, TMTB and AFI, were associated with decrements in GS at enrollment. Both of these tests are reported to measure aspects of the same outcome, executive function, a set of mental skills that involves the prefrontal cortex and includes working memory, cognitive flexibility, and self-control [43]. However, when our patients’ TMTB scores were compared to the general population, our sample’s scores are significantly worse (i.e., 112.5 s vs 99.8 s, respectively, *t* = 2.7, *p* < 0.01) [44]. In contrast, while our sample’s mean AFI scores were relatively high (7.4) [29], 49.1% of the patients had scores in the low (< 5) to moderate (5.0 to 7.5) range. These findings suggest that TMTB may have higher sensitivity and specificity to detect changes in CF in older adults. That said, both measures were retained in the final model which suggests that they measure different aspects of CF and warrant additional evaluation in future studies.

While less well studied than balance and GS, being able to rise from a chair is one of the basic movements in everyday life. The chair-stand test reflects muscle size, strength, and power, in addition to visual contrast sensibility, lower limb proprioception, and peripheral tactile sensitivity [45, 46]. While we identified a large amount of inter-individual variability, our patients’ average chair-stand time at the initiation of chemotherapy (13.4 s) was poorer than an age-matched normative population (10.9 s, *t* = 4.9, *p* < 0.01) [14]. Consistent with a previous study of older patients with leukemia [10], chair-stand improved from initiation of through 12 months after chemotherapy administration.

A lower KPS score, higher BMI, and worse TMTB scores were the characteristics associated with poorer chair-stand times at enrollment. In addition, a worse chair-stand time at enrollment was associated with decrements in the chair-stand test over time. It is interesting to note that like the balance test, lower KPS scores were associated with poorer chair-stand time at enrollment. One plausible explanation for this association is that a KPS score of ≤ 70 is associated with deconditioning and weakness of the muscles in the lower extremities that would interfere with the ability to rise from a chair without upper extremity assistance. Future studies need to evaluate for associations between lower KPS scores and muscle strength and tone.

Patients with a higher BMI had poor chair-stand time at the initiation of chemotherapy. Our sample’s mean BMI of 26.2 is in the overweight range [47]. While no studies identified this association, it is reasonable to assume that patients with a higher BMI would have more difficulty rising from a chair without the assistance. Given that BMI is a modifiable risk factor, patients with a high BMI may warrant referrals for dietary counseling and exercise.

Consistent with a study of community-dwelling older adults [48], impairments in CF were associated with poorer chair-stand times. In addition to muscle strength and balance, chair-stand requires motor coordination and ability to use visuospatial information [49]. Given that reduced CF is associated with poorer mobility [24] and that decrements in executive function and processing speed may affect one’s ability to execute PF tasks, chair-stand times may worsen. Further, patients with reduced CF may be less motivated to maintain physical activity with a resultant decline in PF [49].

Patients with a poorer chair-stand time at initiation of chemotherapy had improvements in their chair-stand time over 12 months. In contrast, patients with a better chair-stand time at enrollment had a slight worsening of their chair-stand time over the same period. Given that 54.5% of the sample had surgery prior to the initiation of chemotherapy, patients with worse chair-stand times may have been recovering from surgery. This relatively simple test can be used by clinicians to evaluate PF in older adults during chemotherapy.

Several limitations warrant consideration. First, given that our sample was predominately women with gynecological cancer, married, and had metastatic disease, our findings may not generalize to all older oncology patients. Second, chemotherapy regimen, nutritional status, and risk for falls that could contribute to declines in PF were not evaluated [1]. While the literature suggests that associations exist between decrements in CF and poorer balance [50], and associations were found in our exploratory analysis, our subjective and objective measures of CF were not retained in the final model. This lack of association may be partially explained by our sample size and warrants evaluation in future studies.

However, the assessment and measurement of changes in three objective measures of PF over a year and the use of HLM to identify characteristics associated with decrements in balance, GS, and chair-stand are major strengths of this study. In addition, this study is the first to evaluate for variations in the trajectories of the three measures, as well as for associations with demographic, clinical, symptom, and CF characteristics. Our findings can help clinicians identify older oncology patients at risk for decrements in PF. Future research needs to develop interventions to evaluate various aspects of PF in older patients with cancer.

## Supplementary Information

Below is the link to the electronic supplementary material.

Supplementary file1 (DOCX 30 KB)

Supplementary file2 (DOCX 22 KB)

## Data Availability

Not applicable.

## References

[1] Cohen HJ, Schmader KE, (2018) Comprehensive geriatric assessment for patients with cancer. MES Reed E Drews, MD, Editor.

[2] Torstveit AH, Løyland B, Grov EK, Guren M, Paul S, Ritchie C (2021). Distinctions between self-report and performance-based measures of physical function in older patients prior to chemotherapy. Cancer Nurs.

[3] Wong ML, Paul SM, Mastick J, Ritchie C, Steinman M, Walter L (2018). Characteristics associated with physical function trajectories in older adults with cancer during chemotherapy. J Pain Symptom Manage.

[4] Miaskowski C, Wong ML, Cooper BA, Mastick J, Paul S, Possin K (2017). Distinct physical function profiles in older adults receiving cancer chemotherapy. J Pain Symptom Manage.

[5] Kirkhus L, Harneshaug M, Šaltytė Benth J, Grønberg BH, Rostoft S, Bergh S (2019). Modifiable factors affecting older patients’ quality of life and physical function during cancer treatment. J Geriatr Oncol.

[6] Hoppe S, Rainfray M, Fonck M, Hoppenreys L, Blanc JF, Ceccaldi J (2013). Functional decline in older patients with cancer receiving first-line chemotherapy. J Clin Oncol.

[7] Hurria A, Hurria A, Zuckerman E, Panageas KS, Fornier M, D'Andrea G (2006). A prospective, longitudinal study of the functional status and quality of life of older patients with breast cancer receiving adjuvant chemotherapy. J Am Geriatr Soc.

[8] Naito T, Okayama T, Aoyama T, Ohashi T, Masuda Y, Kimura M (2017). Skeletal muscle depletion during chemotherapy has a large impact on physical function in elderly Japanese patients with advanced non–small-cell lung cancer. BMC Cancer.

[9] Mohamedali H, Breunis H, Timilshina N, Brandwein JM, Gupta V, Li M (2012). Older age is associated with similar quality of life and physical function compared to younger age during intensive chemotherapy for acute myeloid leukemia. Leuk Res.

[10] Alibhai SM, Breunis H, Timilshina N, Brignardello-Petersen R, Tomlinson G, Mohamedali H (2015). Quality of life and physical function in adults treated with intensive chemotherapy for acute myeloid leukemia improve over time independent of age. J Geriatr Oncol.

[11] Klepin HD, Tooze JA, Pardee TS, Ellis LR, Berenzom D, Mihalko SL (2016). Effect of intensive chemotherapy on physical, cognitive, and emotional health of older adults with acute myeloid leukemia. J Am Geriatrs Soc.

[12] Mian H, Pond GR, Tuchman SA, Fiala MA, Wildes TM (2020). Geriatric assessment and quality of life changes in older adults with newly diagnosed multiple myeloma undergoing treatment. J Geriatr Oncol.

[13] Guralnik JM, Ferrucci L, Pieper CF, Leveille SG, Markides KS, Ostir GV (2000). Lower extremity function and subsequent disability: consistency across studies, predictive models, and value of gait speed alone compared with the short physical performance battery. J Gerontol A.

[14] Bergland A, Strand BH (2019). Norwegian reference values for the Short Physical Performance Battery (SPPB): the Tromsø Study. BMC Geriatr.

[15] Belvederi Murri M, Triolo F, Coni A, Tacconi C, Nerozzi E, Escelsior A (2020). Instrumental assessment of balance and gait in depression: a systematic review. Psychiatry Res.

[16] Hirase T, Okubo Y, Sturnieks DL, Lord SR (2020). Pain is associated with poor balance in community-dwelling older adults: a systematic review and meta-analysis. J Am Med Assoc.

[17] Stijntjes M, Pasma JH, Van Vuuren M, Blauw GJ, Meskers CGM, Maier AB (2015). Low cognitive status is associated with a lower ability to maintain standing balance in elderly outpatients. Gerontol.

[18] Cohen JA, Verghese J, Zwerling JL (2016). Cognition and gait in older people. Maturitas.

[19] Montero-Odasso M, Almeida QJ, Bherer L, Burhan AM, Camicioli R, Doyon J (2019). Consensus on shared measures of mobility and cognition: from the Canadian Consortium on Neurodegeneration in Aging (CCNA). J Gerontol A.

[20] Kyrönlahti SM, Almeida QJ, Bherer L, Burhan AM, Camicioli R, Doyon J (2020). Educational differences in decline in maximum gait speed in older adults over an 11-year follow-up. J Gerontol A.

[21] Cesari M, Cerullo F, Zamboni V, Di Palma R, Scambia G, Balducci L (2013). Functional status and mortality in older women with gynecological cancer. J Gerontol A.

[22] Taylor JL, Parker LJ, Szanton SL, Thorpe RJ (2018). The association of pain, race and slow gait speed in older adults. Geriatr Nurs.

[23] Atkinson HH, Rosano C, Simonsick EM, Williamson JD, Davis C, Ambrosius WT (2007). Cognitive function, gait speed decline, and comorbidities: the Health, Aging and Body Composition Study. J Gerontol A.

[24] Utne I, Loyland B, Grov EK, Rasmussen HL, Torstveit AH, Paul SM, et al (2021) Age-related differences in self-report and objective measures of cognitive function in older patients prior to chemotherapy. Nurs Open. 10.1002/nop2.1141 10.1002/nop2.1141 PMC8859071 34878233

[25] Torstveit AH, Miaskowski C, Løyland B, Grov EK, Guren MG, Ritchie CS (2021). Common and distinct characteristics associated with self-reported functional status in older patients with cancer receiving chemotherapy. Eur J Oncol Nurs.

[26] Nasreddine ZS, Phillips NA, Bédirian V, Charbonneau S, Whitehead V, Collin,  (2005). The Montreal Cognitive Assessment, MoCA: a brief screening tool for mild cognitive impairment. J Am Geriatr Soc.

[27] Schag CC, Heinrich RL, Ganz P (1984). Karnofsky performance status revisited: reliability, validity, and guidelines. J Clin Oncol.

[28] Sangha O, Stucki G, Liang MH, Fossel AH, Katz JN (2003). The self-administered comorbidity questionnaire: a new method to assess comorbidity for clinical and health services research. Arthritis Care Res.

[29] Cimprich B, Visovatti M, Ronis DL (2011). The Attentional Function Index—a self-report cognitive measure. Psychooncol.

[30] Tombaugh TN (2004). Trail Making Test A and B: normative data stratified by age and education. Arch Clin Neuropsychol.

[31] Raudenbush SW, Bryk AS. (2002) Hierarchical linear models: applications and data analysis methods (2nd ed.), Sage Publications, Thousand Oaks, CA

[32] Takacs J, Carpenter MG, Garland SJ, Hunt MA (2013). The role of neuromuscular changes in aging and knee osteoarthritis on dynamic postural control. Aging Dis.

[33] Teo W-P, Goodwill AM, Hendy AM (2018). Sensory manipulation results in increased dorsolateral prefrontal cortex activation during static postural balance in sedentary older adults: an fNIRS study. Brain Behav.

[34] Gewandter JS, Fan L, Magnuson A, Muthalib M, Macpherson H (2013). Falls and functional impairments in cancer survivors with chemotherapy-induced peripheral neuropathy (CIPN): a University of Rochester CCOP study. Support Care Cancer.

[35] Garber CE, Greaney ML, Riebe NCR, Burbank PA, Clark PG (2010). Physical and mental health-related correlates of physical function in community dwelling older adults: a cross sectional study. BMC Geriatr.

[36] Avlund K, Damsgaard MT, Osler M (2004). Social position and functional decline among non-disabled old men and women. Eur J Public Health.

[37] Pinheiro LC, Reshetnyak E, Akinyemiju T, Phillips E, Safford MM (2021) Social determinants of health and cancer mortality in the Reasons for Geographic and Racial Differences in Stroke (REGARDS) cohort study. Cancer. 10.1002/cncr.33894 10.1002/cncr.33894 PMC9301452 34478162

[38] Wilcoxon A, Kober KM, Viele C, Topp K, Smoot B, Abrams G (2020). Association between physical activity levels and chemotherapy-induced peripheral neuropathy severity in cancer survivors. Oncol Nurs Forum.

[39] Duregon F, Vendramin B, Bullo V, Gobbo S, Cugusi L, Di Blasio A (2018). Effects of exercise on cancer patients suffering chemotherapy-induced peripheral neuropathy undergoing treatment: a systematic review. Crit Rev Oncol Hematol.

[40] Ferrucci L, Cooper R, Shardell M, Simonsick EM, Schrack JA, Kuh D (2016). Age-related change in mobility: perspectives from life course epidemiology and geroscience. J Gerontol A.

[41] Wennberg AM, Savica R, Mielke MM (2017). Association between various brain pathologies and gait disturbance. Dement Geriatr Cogn Dis.

[42] Utne I, Cooper BA, Ritchie C, Wong M, Dunn LB, Loyland B (2020). Co-occurrence of decrements in physical and cognitive function is common in older oncology patients receiving chemotherapy. Eur J Oncol Nurs.

[43] Diamond A (2013). Executive functions. Annu Rev Psychol.

[44] MacPherson SE, Cox SR, Dickie DA, Karama S, Starr JM, Evans AC (2017). Processing speed and the relationship between Trail Making Test-B performance, cortical thinning and white matter microstructure in older adults. Cortex.

[45] Yoshiko A, Ogawa M, Shimizu K, Radaelli R, Neske R, Maeda H (2021). Chair sit-to-stand performance is associated with diagnostic features of sarcopenia in older men and women. Arch Gerontol Geriatr.

[46] Alcazar J, Aagaard P, Haddock B, Kamper RS, Hansen SK, Prescott E (2020). Age- and sex-specific changes in lower-limb muscle power throughout the lifespan. J Gerontol A.

[47] World Health Organization (2000). Obesity: preventing and managing the global epidemic in Report of a WHO consultation. World Health Org Tech Rep Series.

[48] Dodds RM, Murray JC, Granic A, Hurst C, Uwimpuhwe G, Richardson S (2021). Prevalence and factors associated with poor performance in the 5-chair stand test: findings from the Cognitive Function and Ageing Study II and proposed Newcastle protocol for use in the assessment of sarcopenia. J Cachexia Sarcopenia Muscle.

[49] Annweiler C, Schott AM, Abellan Van Kan G, Rolland Y, Blain H, Fantino B (2011). The Five-Times-Sit-to-stand test, a marker of global cognitive functioning among community-dwelling older women. J Nutr Health Aging.

[50] Blackwood J (2019) The influence of cognitive function on balance, mobility, and falls in cancer survivors. Rehabil Oncol 37(2) 10.1097/01.REO.0000000000000128

