# Supplementary material for: Characteristics associated with decrements in objective measures of physical function in older patients with cancer during chemotherapy
Source: Support Care Cancer. 2022 Nov 3;30(12):10031–41. doi: 10.1007/s00520-022-07416-5 (PMC9715479; doi:10.1007/s00520-022-07416-5)
Supplement: Supplementary file 1 — Supplementary file1 (DOCX 30 KB) [file 520_2022_7416_MOESM1_ESM.docx]

**METHODS**

**Sample and Settings**

Eligible patients were ≥60 years of age; had a diagnosis of gynecological or colorectal cancer; were scheduled to receive primary or adjuvant chemotherapy; had a Montreal Cognitive Assessment (MoCA) score of ≥23;[1] and had a Karnofsky Performance Status (KPS) score of ≥60.[2] A total of 208 patients were approached and 149 consented to participate (71.6% response rate). Of these 149 patients, one withdrew and nine were excluded because they had a MoCA score of <23. For this analysis, complete data from 112 patients were available.

**Instruments**

*Demographic and clinical characteristics* - Patients completed a demographic questionnaire, the KPS scale,[3] and the Self-Administered Comorbidity Questionnaire (SCQ-16).[4] The SCQ-16 evaluates the occurrence of, treatments for, and functional impact of 16 common comorbid conditions. Total SCQ scores can range from 0 to 48.

*Objective measures of PF –* Three measures from the SPPB (i.e., balance, gait speed, chair-stand) were used to assess PF.[5] For the balance test, patients had to stand with their feet in side-by-side, semi-tandem, and tandem positions without assistance. Patients were asked to hold each position for ten seconds (sec). Based on established cutpoints, all of these tests were scored on a 0-1, 0-1, and 0-2 scale, respectively.[6] A total score of ≥3.71 (±0.65) is considered normal.[7]

Gait speed was scored based on the time taken to walk 3 or 4 meters at usual speed. The test was performed twice, and the shortest time was used in the analysis. Tape was used to mark out the distance on a flat unobstructed course. Patients began in a standing position, with their toes just touching the start line. The timer was started when the patients began moving and was stopped when the patients` foot completely crossed the 3- or 4-meter line. Completion times of <3.62 or <4.82 sec (i.e., <1.2 meter/second (m/sec)) respectively, are considered normal.[7]

The repeated chair-stand test was scored as the time taken to complete five repetitions of the sit-to-stand maneuver. All sit-to-stand maneuvers were performed using a dining chair. Patients were instructed to fold their arms across their chest during the test. The timer was started when the patients` back left the backrest and was stopped when they straightened out completely for the fifth time. A completion time of ≤11.19 sec is considered normal.[7]

*Symptom measures* *-* The 20-item Center for Epidemiological Studies-Depression (CES-D) scale was used to evaluate depressive symptoms.[8] Total score can range from 0 to 60, with scores of ≥16 indicating the need for individuals to seek clinical evaluation for major depression. In the current study, the Cronbach`s alpha for the CES-D total score was 0.84.

The European Organization for the Research and Treatment of Cancer Quality of Life Questionnaire Core-30 (i.e., QLQ-C30) was the measure of additional symptoms in this study. It consists of eight symptom scales (i.e., fatigue, pain, nausea and vomiting, dyspnea, insomnia, appetite loss, constipation, diarrhea).[9] The raw scores were linearly transformed to a 0 to 100 scale, using the algorithm in the QLQ-C30 scoring manual.[10] Higher scores indicate more severe symptoms. In the current study, the Cronbach`s alpha for the fatigue subscale that had more than 2 items was 0.90.

*Subjective measure of CF* – The 16-item Attentional Function Index (AFI) assesses an individual`s perceived effectiveness in performing daily activities that are supported by attention and working memory.[11, 12] Each item was rated on a 0 to 10 numeric rating scale and a total score was calculated as the mean of these items. A higher total AFI score indicates greater capacity to direct attention.[11] Total scores are grouped into three categories of attentional function (i.e.,<5.0 low function, 5.0 to 7.5 moderate function, >7.5 high function).[13] In the current study, its Cronbach’s alpha was 0.93.

*Objective measures of CF* - The MoCA is a brief screening tool designed to detect mild forms of cognitive impairment.[14] It evaluates six cognitive domains: memory, visuospatial abilities, executive functioning, attention and concentration, language, and orientation.[1] If an individual`s education is ≤12 years, 1 point is added to the total score to achieve a maximum score of 30.[1] Scores of ≤25 indicate the presence of cognitive impairment.[1] MoCA has good psychometric properties, is sensitive to change over time,[15] and was used in studies of older oncology patients.[16-18]

Trial Making Test B (TMTB) provides information on difficulties with executive function (i.e., mental flexibility, including task switching, shared attention, working memory, simultaneous capacity, and planning).[19] TMTB requires an individual to draw lines sequentially alternating between connecting 13 numbers and 13 letters (e.g., 1, A, 2, B, 3, C,…13, L). The score is the amount of time required to complete the task.[19, 20] TMTB does not have established cut-off scores.[19] The TMT is a valid measure to assess CF in older patients [20] and was used in studies of older oncology patients.[21, 22]

**Study procedures**

Regional Committee for Medical and Research Ethics, Norway, and the Institutional Review Board at each of the study sites approved the study (reference No. 2015/1277/REC South East). Oncologists or nurses approached patients prior to the initiation of chemotherapy to assess their interest in study participation. Written informed consent was obtained from all patients. Patients completed study questionnaires and the PF tests in their homes or in the clinic, a total of six times over one year (i.e., at the initiation of and approximately one, three, six, nine, and twelve months after chemotherapy administration). Reliability testing for all of the study measures was done on an annual basis with all of the research staff. An inter-rater reliability of >.90 was achieved for all the study measures.

**Statistical Analysis**

Descriptive statistics were generated for demographic and clinical characteristics, symptom severity scores, and measures of physical function (PF) and cognitive function (CF) using SPSS version 26 (IBM Corporation, Armonk, NY). All of the demographic, clinical, and symptom characteristics, as well as the CF measures that were evaluated as predictors in the hierarchical linear modeling (HLM) analysis were assessed at the initiation of chemotherapy.

As described previously, [23-25] HLM based on full maximum likelihood estimation was done using the software developed by Raudenbush and colleagues.[26]. Separate HLM analyses were done for the balance, gait speed, and chair-stand tests. In brief, during stage 1, intra-individual variability in the scores for each of the tests over time was examined. At this point, the model was constrained to be unconditional and likelihood ratio tests were used to determine the best fitting model.

Second stage of HLM analysis examined inter-individual differences in the trajectories of each of the test’s scores by modeling the individual change parameters as a function of proposed predictors at level 2. Supplemental Tables 1, 2, and 3 present the list of proposed predictors for the balance, gait speed, and chair-stand tests, respectively, that was developed based on a literature review on PF in older oncology patients.[27-30]

To improve estimation efficiency and construct a parsimonious model, an exploratory level 2 analysis was completed in which each potential predictor was assessed to determine whether it would result in a better model if it alone was added as a level 2 predictor. Predictors with a t-value of <2.0 were dropped from subsequent model testing. All potential significant predictors from the exploratory analyses were entered into the model to predict each individual change parameter. Only predictors that maintained a statistically significant contribution in conjunction with other variables were retained in the final model. A *p*-value of <.05 indicated statistical significance.

**References**

1. Nasreddine ZS, Phillips NA, Bédirian V, Charbonneau S, Whitehead V, Collin I, et al. The Montreal Cognitive Assessment, MoCA: A brief screening tool for mild cognitive impairment. J Am Geriat Soc 2005; 53: 695-9. <https://doi.org/10.1111/j.1532-5415.2005.53221.x>

2. Schag CC, Heinrich RL, Ganz P. Karnofsky performance status revisited: reliability, validity, and guidelines. J Clin Oncol 1984; 2: 187-93.

3. Schnadig ID, Fromme EK, Loprinzi CL, Sloan JA, Mori M, Li H, et al. Patient‐physician disagreement regarding performance status is associated with worse survivorship in patients with advanced cancer. Cancer 2008; 113: 2205-14. <https://doi.org/10.1002/cncr.23856>

4. Sangha O, Stucki G, Liang MH, Fossel AH, Katz JN, The self-administered comorbidity questionnaire: A new method to assess comorbidity for clinical and health services research. Arthritis Care Res 2003; 49: 156-63. <https://doi.org/10.1002/art.10993>

5. Studenski S, Perera S, Wallace D, Chandler JM, Duncan PW, Rooney E, Fox M, et al. Physical performance measures in the clinical setting. J Am Geriatr Soc 2003; 51: 314-22. <https://doi.org/10.1046/j.1532-5415.2003.51104.x>

6. Guralnik JM, Simonsick EM, Ferrucci L, Glynn RJ, Berkman LF, Blazer DG, et al. A Short Physical Performance Battery assessing lower extremity function: Association with self-reported disability and prediction of mortality and nursing home admission. J Gerontol 1994; 49: M85-M94. <https://doi.org/10.1093/geronj/49.2.M85>

7. Bergland A, Strand BH. Norwegian reference values for the Short Physical Performance Battery (SPPB): the Tromsø Study. BMC Geriatr 2019; 19: 216. <https://doi.org/10.1186/s12877-019-1234-8>

8. Radloff L. A self-report depression scale for research in the general population. Appl Psychol Meas 1977; 1: p. 385-401.

9. Aaronson NK, Ahmedzai S, Bergman B, Bullinger M, Cull A, Duez NJ, et al. The European Organization for Research and Treatment of Cancer QLQ-C30: A quality-of-life instrument for use in international clinical trials in oncology. JNCI Cancer Spectr 1993; 85: 365-76. h[ttps://doi.org/10.1093/jnci/85.5.365](https://doi.org/10.1093/jnci/85.5.365)

10. Fayers P, Aaronson N, Bjordal K, Groenvold M, Curran D, Bottomly A, European Organisation for Research and Treatment of Cancer QLQ-C30 scoring manual, ed 3rd edn. EORTC Quality of Life Group, Brussels 2001.

11. Cimprich B, Visovatti M, Ronis DL. The Attentional Function Index—A self‐report cognitive measure. Psychooncology 2011; 20: 194-202. <https://doi.org/10.1002/pon.1729>

12. Utne I, Grov EK, Kjerland LE, Rønning C, Rodrigues-Aranda C, Rasmussen HL, et al. Translation and cultural adaptation of the Attentional Function Index (AFI)(Oversettelse of kulturell tilpasning av Attentional Function Index (AFI)). Sykepleien forskning 2017; 12.

13. Cimprich B, So H, Ronis DL, Trask C. Pre-treatment factors related to cognitive functioning in women newly diagnosed with breast cancer. Psychooncology 2005; 14: 70-8. <https://doi.org/10.1002/pon.821>

14. Dong Y, Lee WY, Basri NA, Collinson SL, Merchant RA, Venketasubramanian N, et al. The Montreal Cognitive Assessment is superior to the Mini–Mental State Examination in detecting patients at higher risk of dementia. Int Psychogeriatr 2012; 24: 1749-55. <https://doi.org/10.1017/S1041610212001068>

15. Freitas S, Simões MR, Marôco J, Alves L, Santana I. Construct validity of the Montreal Cognitive Assessment (MoCA). J Int Neuropsychological Soc 2012; 18: 242-50. <https://doi.org/10.1017/S1355617711001573>

16. Carlson BW, Craft MA, Carlson JR, Razaq W, Deardeuff KK, Benbrook DM. Accelerated vascular aging and persistent cognitive impairment in older female breast cancer survivors. GeroScience 2018; 40: 325-36. <https://doi.org/10.1007/s11357-018-0025-z>

17. Loh KP, Pandya C, Zittel J, Kadambi S, Flannery M, Reizine N, et al. Associations of sleep disturbance with physical function and cognition in older adults with cancer. Sup Care Cancer 2017; 25: 3161-69. <https://doi.org/10.1007/s00520-017-3724-6>

18. Edwards BJ, Zhang X, Sun M, Holmes HM, Ketonen L, Guha N, et al. Neurocognitive deficits in older patients with cancer. J Geriatr Oncol 2018; 9: 482-7. <https://doi.org/10.1016/j.jgo.2018.02.010>

19. Tombaugh TN. Trail Making Test A and B: Normative data stratified by age and education. Arch Clin Neuropsychol 2004; 19: 203-14. <https://doi.org/10.1016/S0887-6177(03)00039-8>

20. Reitan RM. Validity of the Trail Making Test as an indicator of organic brain damage. Percept Mot Skills 2016; 8: 271-6.

21. Khan OF, Cusano E, Raissouni S, Pabia M, Haeseker J, Bosma N, et al. Immediate-term cognitive impairment following intravenous (IV) chemotherapy: a prospective pre-post design study. BMC cancer 2019; 19: 1-9. <https://doi.org/10.1186/s12885-019-5349-2>

22. Weerink LB, van Leeuwen BL, Gernaat SA, Absalom AR, Huisman MG, Van der Wal-Huisman H, et al. Vitamin status and the development of postoperative cognitive decline in elderly surgical oncologic patients. Ann Surg Oncol 2018; 25: 231-8. <https://doi.org/10.1245/s10434-017-6118-6>

23. Miaskowski C, Paul SM, Cooper BA, Lee K, Dood M, West C, et al. Trajectories of fatigue in men with prostate cancer before, during, and after radiation therapy. J Pain Symptom Manage 2008; 35: 632-43. <https://doi.org/10.1016/j.jpainsymman.2007.07.007>

24. Dhruva A, Dodd M, Paul SM, Cooper BA, Lee K, West C, et al. Trajectories of fatigue in patients with breast cancer before, during, and after radiation therapy. Cancer Nurs 2010; 33: 201-12. https://doi.org/[10.1097/NCC.0b013e3181c75f2a](https://dx.doi.org/10.1097%2FNCC.0b013e3181c75f2a)

25. Miaskowski C, Paul SM, Cooper BA, Lee K, Dodd M, West C, et al. Predictors of the trajectories of self-reported sleep disturbance in men with prostate cancer during and following radiation therapy. Sleep 2011; 34: 171-79. <https://doi.org/10.1093/sleep/34.2.171>

26. Raudenbush SW, Bryk AS. Hierarchical linear models: Applications and data analysis methods. sage,2002.

27. Wong ML, Paul SM, Mastick J, Ritchie C, Steinman MA, Walter LC, et al. Characteristics associated with physical function trajectories in older adults with cancer during chemotherapy. J Pain Symptom Manage 2018; 56: 678-88. e671. <https://doi.org/10.1016/j.jpainsymman.2018.08.006>

28. Miaskowski C, Wong ML, Cooper BA, Mastick J, Paul SM, Possin K, et al. Distinct physical function profiles in older adults receiving cancer chemotherapy. J Pain Symptom Manage 2017; 54: 263-72. <https://doi.org/10.1016/j.jpainsymman.2017.07.018>

29. Kirkhus L, Harneshaug M, Šaltytė Benth J, Grønberg BH, Rosthoft S, Bergh S, et al. Modifiable factors affecting older patients' quality of life and physical function during cancer treatment. J Geriatr Oncol 2019; 10: 904-12. <https://doi.org/10.1016/j.jgo.2019.08.001>

30. Hoppe S, Rainfray M, Fonck M, Hoppenreys L, Blanc JF, Ceccaldi J, et al. Functional decline in older patients with cancer receiving first-line chemotherapy. J Clin Oncol 2013; 31: 3877-82.
