# Supplementary material for: Characteristics associated with decrements in objective measures of physical function in older patients with cancer during chemotherapy
Source: Support Care Cancer. 2022 Nov 3;30(12):10031–41. doi: 10.1007/s00520-022-07416-5 (PMC9715479; doi:10.1007/s00520-022-07416-5)
Supplement: Supplementary file 2 — Supplementary file2 (DOCX 22 KB) [file 520_2022_7416_MOESM2_ESM.docx]

**Appendix B**

Supplemental table 1A – Potential predictors of the intercept (I) and linear coefficient (LC), and quadratic coefficient (QC) for the balance test

| Characteristics | I | LC | QC |
| --- | --- | --- | --- |
| Demographic characteristics | | | |
| Age in years | X | X | X |
| Female gender |  |  |  |
| Lives alone |  |  |  |
| Marital status |  |  |  |
| Currently employed | X | X | X |
| Education | X | X | X |
| Clinical characteristics | | | |
| Cancer diagnosis |  |  |  |
| Time since cancer diagnosis |  |  |  |
| Presence of metastatic disease |  |  |  |
| Surgery prior to chemotherapy | X | X | X |
| Body mass index |  |  |  |
| Karnofsky Performance Status score | X | X | X |
| Number of comorbidities |  |  |  |
| Self-administered Comorbidity Questionnaire score | X | X | X |
| Hemoglobin (grams/deciliter) |  |  |  |
| Symptom severity scores | | | |
| Depression |  |  |  |
| Fatigue | X | X | X |
| Nausea and vomiting |  |  |  |
| Insomnia |  |  |  |
| Appetite loss |  |  |  |
| Constipation |  |  |  |
| Diarrhea |  |  |  |
| Pain |  |  |  |
| Dyspnea |  |  |  |
| Physical and cognitive function measures | | | |
| Balance test score | ---- | X | X |
| Attentional Function Index score | X | X | X |
| Montreal Cognitive Assessment score | X | X | X |
| Trail Making Test B (seconds) | X | X | X |

Supplemental table 1B – Potential predictors of the intercept (I) and linear coefficient (LC) for gait speed

| Characteristics | I | LC |
| --- | --- | --- |
| Demographic characteristics | | |
| Age in years | X |  |
| Female gender | X |  |
| Lives alone |  |  |
| Marital status |  |  |
| Currently employed | X |  |
| Education | X |  |
| Clinical characteristics | | |
| Cancer diagnosis | X |  |
| Time since cancer diagnosis |  |  |
| Presence of metastatic disease |  |  |
| Surgery prior to chemotherapy |  |  |
| Body mass index |  |  |
| Karnofsky Performance Status score |  |  |
| Number of comorbidities |  |  |
| Self-administered Comorbidity Questionnaire score |  |  |
| Hemoglobin (grams/deciliter) |  |  |
| Symptom severity scores | | |
| Depression | X |  |
| Fatigue |  |  |
| Nausea and vomiting |  |  |
| Insomnia |  |  |
| Appetite loss |  |  |
| Constipation | X |  |
| Diarrhea |  |  |
| Pain |  | X |
| Dyspnea |  |  |
| Physical and cognitive function measures | | |
| Gait speed at enrollment (meters per second) | ---- | X |
| Attentional Function Index score | X |  |
| Montreal Cognitive Assessment score | X |  |
| Trail Making Test B (seconds) | X |  |

Supplemental table 1C – Potential predictors of the intercept (I) and linear coefficient (LC) for the chair stand test

| Characteristics | I | LC |
| --- | --- | --- |
| Demographic characteristics | | |
| Age in years |  |  |
| Female gender | X |  |
| Lives alone |  |  |
| Marital status |  |  |
| Currently employed | X |  |
| Education | X |  |
| Clinical characteristics | | |
| Cancer diagnosis | X |  |
| Time since cancer diagnosis |  |  |
| Presence of metastatic disease |  |  |
| Surgery prior to chemotherapy |  |  |
| Body mass index | X |  |
| Karnofsky Performance Status score | X |  |
| Number of comorbidities | X |  |
| Self-administered Comorbidity Questionnaire score | X |  |
| Hemoglobin (grams/deciliter) |  |  |
| Symptom severity scores | | |
| Depression | X |  |
| Fatigue | X |  |
| Nausea and vomiting |  |  |
| Insomnia |  |  |
| Appetite loss | X |  |
| Constipation |  |  |
| Diarrhea | X |  |
| Pain | X |  |
| Dyspnea |  |  |
| Physical and cognitive function measures | | |
| Chair stand test score (seconds) | ---- | X |
| Attentional Function Index score | X |  |
| Montreal Cognitive Assessment score |  |  |
| Trail Making Test B (seconds) | X |  |
